# High and Heterogeneous Prevalence of Asymptomatic and Sub-microscopic Malaria Infections on Islands in Lake Victoria, Kenya

**DOI:** 10.1038/srep36958

**Published:** 2016-11-14

**Authors:** Zulkarnain Md Idris, Chim W. Chan, James Kongere, Jesse Gitaka, John Logedi, Ahmeddin Omar, Charles Obonyo, Beatrice Kemunto Machini, Rie Isozumi, Isao Teramoto, Masatsugu Kimura, Akira Kaneko

**Affiliations:** 1 Island Malaria Group, Department of Microbiology, Tumor and Cell Biology, Karolinska Institutet, Stockholm, 17177, Sweden; 2 Department of Parasitology and Medical Entomology, Faculty of Medicine, Universiti Kebangsaan Malaysia Medical Centre, Kuala Lumpur, 56000, Malaysia; 3 Nagasaki University Nairobi Research Station, NUITM-KEMRI Project, Nairobi, 00202, Kenya; 4 Department of Clinical Medicine, Mount Kenya University, Thika, 01000, Kenya; 5 National Malaria Control Programme, Ministry of Public Health and Sanitation, Nairobi, 00100, Kenya; 6 Kenya Medical Research Institute (KEMRI), Centre for Global Health Research, Kisumu, 40100, Kenya; 7 Department of Parasitology, Graduate School of Medicine, Osaka City University, Osaka, 558-8585, Japan; 8 Institute of Tropical Medicine, Nagasaki University, Nagasaki, 852-8102, Japan

## Abstract

Kenya is intensifying its national efforts in malaria control to achieve malaria elimination. Detailed characterization of malaria infection among populations living in the areas where the disease is endemic in Kenya is a crucial priority, especially for planning and evaluating future malaria elimination strategy. This study aimed to investigate the distribution and extent of malaria infection on islands in Lake Victoria of Kenya to aid in designing new interventions for malaria elimination. Five cross-sectional surveys were conducted between January 2012 and August 2014 on four islands (Mfangano, Takawiri, Kibuogi and Ngodhe) in Lake Victoria and a coastal mainland (Ungoye). Malaria prevalence varied significantly among settings: highest in Ungoye, followed by the large island of Mfangano and lowest in the three remaining small islands. Of the 3867 malaria infections detected by PCR, 91.8% were asymptomatic, 50.3% were sub-microscopic, of which 94% were also asymptomatic. We observed geographical differences and age dependency in both proportion of sub-microscopic infections and asymptomatic parasite carriage. Our findings highlighted the local heterogeneity in malaria prevalence on islands and a coastal area in Lake Victoria, and provided support for the inclusion of mass drug administration as a component of the intervention package to eliminate malaria on islands.

Renewed international commitment to control malaria through scale-up of coverage of interventions reduced the prevalence of *Plasmodium falciparum* by half and the incidence of clinical disease by 40% in endemic Africa between 2000 and 2015^1^. Nevertheless, there were an estimated 188 million cases and 395,000 deaths due to malaria in Africa in 2015. About 73.9% of all malaria deaths in Africa occurred in children before their fifth birthdays^2^. In Kenya, over 31 million people (70.2% of population) are currently at risk of malaria. Malaria accounts for 30% of all outpatient attendance, 19% of hospital admission, and 3–5% of inpatients deaths^3^. Most of the malaria cases in Kenya are due to *P. falciparum*^2^.

Kenya implemented a national strategic malaria control plan in 2001^4^. In recent years aggressive efforts to scale up control measures such as artemisinin-based combined therapy (ACT), rapid diagnostic test (RDT) and long-lasting insecticide net (LLIN) have reduced malaria transmission intensity in most part of the country. Now, 65% (26 million) of Kenyans live in areas where *P. falciparum* parasite rate for the population aged 2–10 years old (P*f*PR_2–10_) is below 1%. However, despite these control tools, 10.6% (4.3 million) of the population still live in areas with P*f*PR_2–10_ ≥ 40%, including areas around Lake Victoria in western Kenya^5^. Kenya launched a revised national malaria strategy for the period 2009–2018. The revised strategy has an increased scope compared to its predecessor with an ambitious vision for a “***malaria free Kenya***”^6^.

Lake Victoria is the second largest fresh water lake in the world, with a surface area of 69,485 km^2^ and a surface altitude of 1,135 m above the sea level. In Homa Bay County, Kenya there are two large islands in Lake Victoria namely Rusinga (east) and Mfangano (west) as well as few other small inhabited islands. Given the year-round availability of fresh water, a high density of malaria vectors is found on these islands, as well as along the lakeshore communities^7^,^8^. Compared to Rusinga Island^9^,^10^ and some mainland areas in the Lake Victoria basin where epidemiological studies have been conducted^11^, there is a dearth of information on malaria endemicity on Mfangano and surrounding small islands. In response, this cross-sectional study aimed to determine the prevalence and geographical distribution of malaria infections among populations on islands in Lake Victoria, Homa Bay County, Kenya. Results from this study provide baseline data for the ongoing plan to implement a malaria elimination package on islands and part of the mainland, where a short-term mass drug administration with ACT plus a small dose of primaquine is combined with sustained vector control with community engagement.

## Results

### Study population

The demographic characteristics of each survey are given in Supplementary Table S1. Except for the survey in March 2014, all other surveys included the five study sites and >2000 participants. Population coverages varied among study sites and among surveys: 9–21% in Ungoye, 4–6% on Mfangano Island and up to 86% on the small islands. On Mfangano, population coverages were 9–42%, 20–62%, 20–38% in the eastern, highland, and western areas, respectively. The gender and age profiles of the participants were representative of the Lake Victoria population as compared to the Nagasaki University-Mbita Health and Demographic Surveillance System (HDSS) and the 2009 Kenyan national census data, with a slight majority of females (52.2%) and a predominance of young individuals (median age 10 years). The majority of the participants were 15 years old and younger (71.9%; 95% confidence interval (CI): 71.0–72.8) and came from the islands (73.0%; 95% CI: 72.2–73.9).

### Prevalence of fever, anaemia, and enlarged spleen

The clinical characteristics of each survey are given in Supplementary Table S1. Of all participants, 579 (5.5%; 95% CI: 5.1–6.0) had a fever and 2297 (22.0%; 95% CI: 21.1–22.8) had a haemoglobin measurement <11 g/dL. Of all children 12 years and below, 2554 (38.9%; 95% CI: 37.7–40.1) were found to have an enlarged spleen with an overall AES index of 1.69. The prevalence of enlarged spleen varied significantly by geographical areas (P < 0.001) and was highest in Ungoye (60.7%; 95% CI: 58.5–62.8, AES = 1.79), followed by Mfangano (36.5%; 95% CI: 34.5–38.6, AES = 1.67) and three small islands (21.1%; 95% CI: 19.4–22.9, AES = 1.50).

### Prevalence of malaria infection

The prevalence of malaria microscopy and PCR in the five surveys is shown in Table 1. Across surveys, parasite rates were 14.6–24.1% by microscopy and 30.1–44.2% by PCR. The overall prevalence was significantly higher (P < 0.05) in male than in female participants by both methods of detection. Parasite rates differed significantly (P < 0.001) by age groups: highest in the 6–10 group by microscopy and 11–15 group by PCR, and lowest in the >30 group by both methods. Malaria prevalence also differed significantly (P < 0.001) among the five studied areas: highest in Ungoye, followed by Mfangano, and lowest on small islands of Takawiri, Kibuogi, and Ngodhe. Geographic heterogeneity in malaria prevalence was consistent among the five surveys.

**Figure 1:**
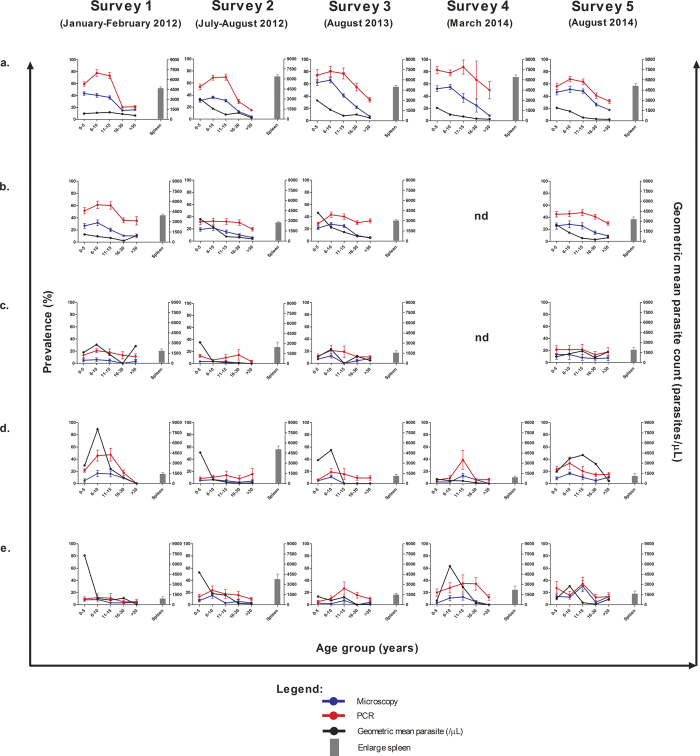
Age trends of malaria prevalence, geometric parasite density and enlarged spleen among children <12 years age in all geographical areas by survey. (**a**) Ungoye, (**b**) Mfangano Island, (**c**) Takawiri Island, (**d**) Kibuogi Island, and (**e**) Ngodhe Island. Error bars represent 95% confidence intervals. nd = not done.

Figure 1 shows detailed results of age-specific prevalence of malaria infection and parasite count among participants in five different geographical areas, and enlarged spleen in five surveys. The peaks in malaria prevalence among the 6–10 and 11–15 groups were most apparent in Ungoye and to a lesser extent Mfangano, but were observed only occasionally on the small islands. Parasite densities were highest among children aged ≤5 years (geometric mean 2023 parasite/μl; 95% CI: 1736.3–2357.3) and decreased significantly (P < 0.001) in older age groups (Supplementary Table S2). Nonetheless, overall parasite densities were similar among geographical areas (P = 0.589) (Supplementary Table S2). Similar to parasite rates, spleen rates among children were consistently highest in Ungoye. Of the 2835 malaria-infected children detected by PCR, 64.3% (95% CI: 62.5–66.1) had an enlarged spleen compared to 19.5% (95% CI: 18.1–20.8) uninfected children (P < 0.001). Furthermore, of the 3865 malaria-infected individuals detected by PCR, 24.8% (95% CI: 23.4–26.2) had anaemia compared to 20.3% (95% CI: 19.4–21.3) of uninfected individuals (P < 0.001).

**Figure 2:**
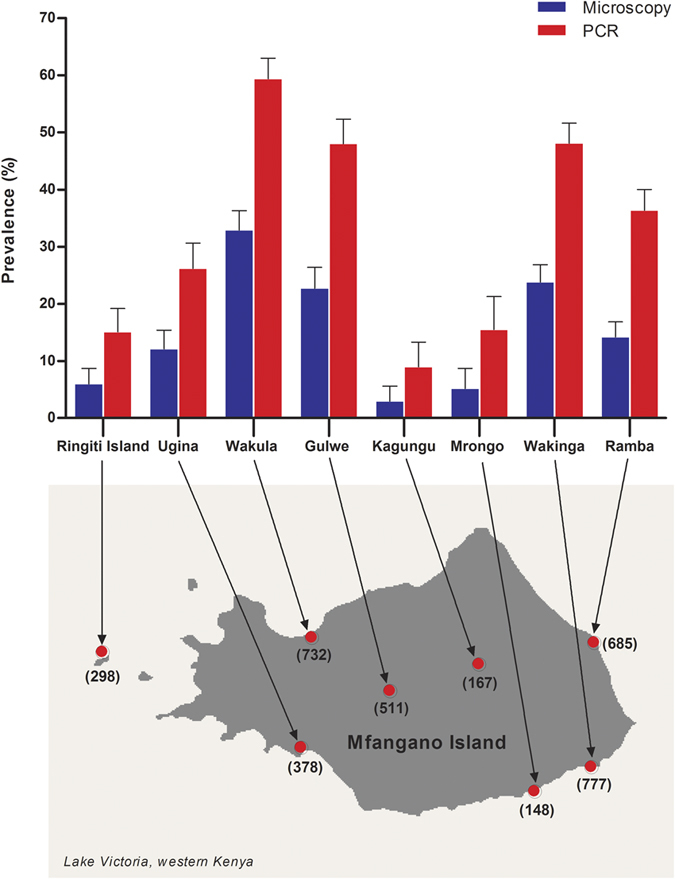
Area-specific prevalence of malaria infection by microscopy and PCR on Mfangano and Ringiti Islands. A number in parentheses is the number of overall sample size. Error bars represent 95% confidence intervals. The map was created with ArcGIS software, version 10.4, http://www.esri.com.

Significant (P < 0.001) intra-island variation in malaria prevalence was also observed among the eight catchment areas on Mfangano Island (Fig. 2). Prevalence by PCR was highest in Wakula (59.4%; 95% CI: 55.8–63.0, n = 435) and lowest in Kagungu (9.0%; 95% CI: 5.1–14.4, n = 15). In the highland area, parasite rates in Gulwe (approximately 100 m above lake level) were significantly higher (P < 0.001) than those in Kagungu (400 m).

**Figure 3:**
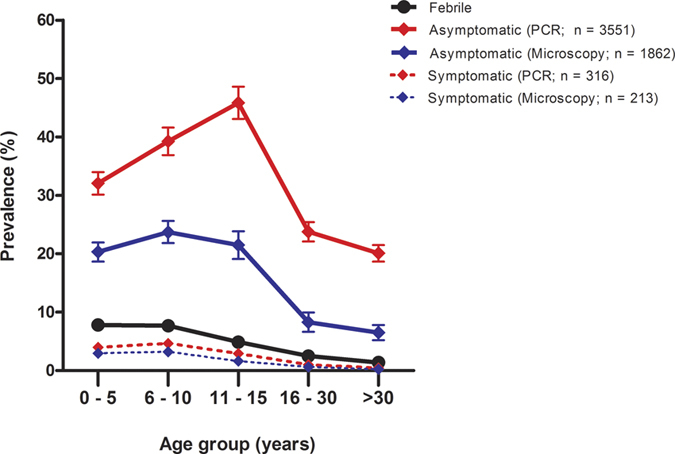
Age trends of febrile illness, asymptomatic and symptomatic malaria infection by microscopy and PCR in Lake Victoria. Error bars represent 95% confidence intervals.

**Figure 4:**
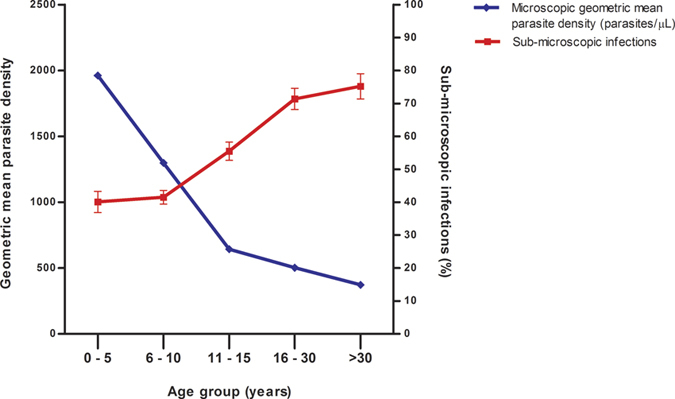
Relationship between proportion of sub-microscopic and parasite density among infected individual in Lake Victoria. Error bars represent 95% confidence intervals.

The majority of positive cases (89.7% by microscopy and 91.8% by PCR) were not accompanied by fever (i.e. asymptomatic) (Fig. 3). The parasite density in symptomatic individuals (geometric mean 3188 parasite/μl, 95% CI: 2430.2–4181.2) were significantly higher than in asymptomatic (geometric mean 1014 parasite/μl, 95% CI: 939.8–1093.7) by microscopy (P < 0.001). Over half (50.4%; 1946/3861) of all malaria infections detected by PCR were sub-microscopic (PCR positive but microscopy negative), and 94% (95% CI: 92.8–95.0) of them were also asymptomatic. The proportion of sub-microscopic infections significantly increased with age (P < 0.001), from 40.1% (95% CI: 36.9–43.4) in children ≤5 years to 75.2% (95% CI: 69.7–80.1) in adults ≥30 years. Furthermore, parasite density assessed by microscopy show a clear age trend of decreasing with increasing sub-microscopic infections (Fig. 4). Interestingly, the proportions of sub-microscopic infections were lowest in the mainland area, moderate in the large island and highest in the small islands (Supplementary Table S3), although parasite densities were not different among geographical areas (Supplementary Table S2).

### Prevalence of *Plasmodium* spp. Infection

Overall, 2076 individuals (19.9%; 95% CI: 19.1–20.7) had *Plasmodium* spp. parasites detectable by microscopy and 3869 participants (37.1%; 95% CI: 36.2–38.0) by PCR (Table 2). Species distribution included a majority of *P. falciparum* mono-infections. *Plasmodium malariae* and *Plasmodium ovale* were less common and no *Plasmodium vivax* infections were observed. PCR detected more mixed-species infections than microscopy. Of the 1176 mixed-species cases detected by PCR, 77.7% (95% CI: 75.2–80.1, n = 914) were double co-infections, contributed mainly by *P. falciparum*/*P. malariae* (n = 760). *P. falciparum*/*P. malariae*/*P. ovale* triple co-infections accounted for 22.3% (95% CI: 19.9–24.8, n = 262) of mixed-species infections. The species-specific prevalence by microscopy and PCR was as follows: 19.7% vs. 33.4% for *P. falciparum*, 1.3% vs. 9.6% for *P. malariae*, and 0.3% vs. 4.0% for *P. ovale*.

**Figure 5:**
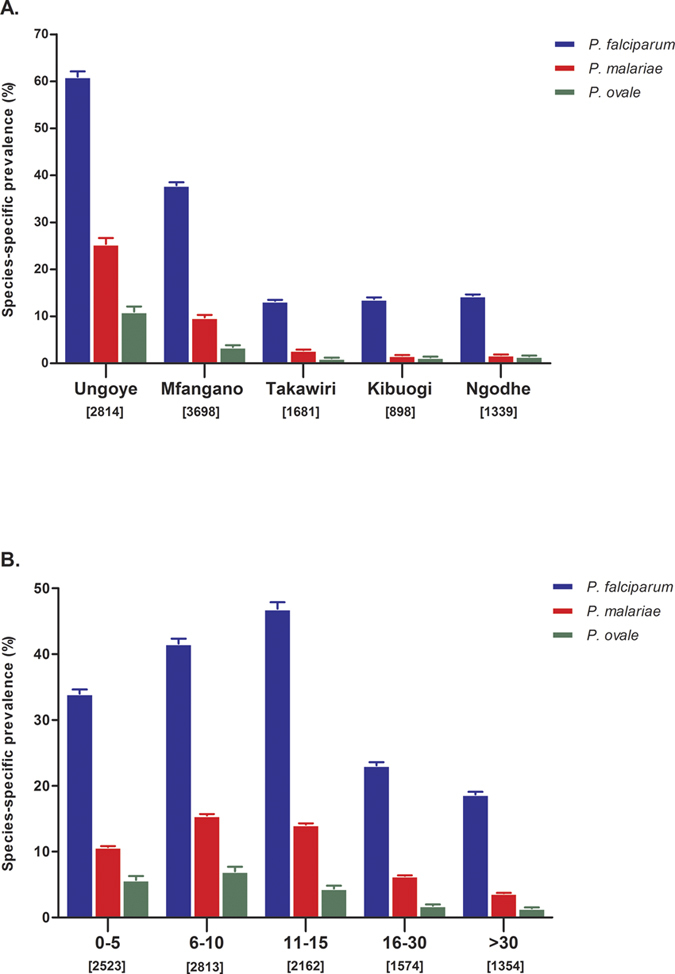
Overall *Plasmodium* spp. prevalence detected by PCR. (**A**) Area-specific prevalence of *Plasmodium* spp., and (**B**) Age-specific prevalence of *Plasmodium* spp. in Lake Victoria. Error bars represent 95% confidence intervals.

The prevalence of *Plasmodium* spp. infections by PCR in different geographical settings and age groups is shown in Fig. 5A,B, respectively. Species-specific rates for all three species were highest in Ungoye, followed by Mfangano and lowest on small islands (Fig. 5A). Species-specific rates were similar among small islands: 13.3–14.4% for *P. falciparum*, 1.7–2.8% for *P. malariae*, and 1.1–1.5% for *P. ovale*. Furthermore, prevalence rates of all three species were age-dependent (P < 0.001) and lowest in >30 years (1.4–18.7%) (Fig. 5B). *P. falciparum*-specific prevalence was highest in the 11–15 group (46.6%; 95% CI: 44.8–49.1, n = 1015). In contrast, *P. malariae*- and *P. ovale*-specific prevalence peaked in children 6–10 years of age, at 15.5% (95% CI: 14.2–16.9, n = 437) and 7.0% (95% CI: 6.1–8.0, n = 198) respectively.

### *P. falciparum* gametocyte prevalence

Among 2054 *P. falciparum* infections detected by microscopy, 10.3% (95% CI: 9.0–11.7, n = 211) had observable gametocytes, resulting in a population *P. falciparum* gametocyte prevalence of 2.02% (95% CI: 1.8–2.3) (Table 2). The proportion of gametocyte positive in *P. falciparum* infections, defined as the percentage of gametocytaemic malaria carriers, was highest in 0–5 age group (13.9%; 95% CI: 11.1–17.0, n = 80) and decreasing significantly (P < 0.001) with age (Table 3). Nonetheless, the proportions of gametocyte positive in *P. falciparum* infections were similar among geographical areas: 10.0% (95% CI: 8.3–11.9) in Ungoye, 10.4% (95% CI: 8.2–12.9) on Mfangano, and 11.2% (95% CI: 7.5–15.9) on the three small islands (Table 3). Among asymptomatic *P. falciparum* infections detected by microscopy, the proportion of gametocyte positive was 10.2% (95% CI: 8.8–11.6).

### Univariable and multivariable associations with malaria infection

As the more sensitive diagnostic method, PCR results were used as the primary outcome of *Plasmodium* infection (with any species) for the risk factor analysis (Table 4). Using the full statistical model, the univariate analyses showed that including season, gender, age group, geographical area, fever case, enlarged spleen and anaemia case significantly improved the fit of the model (all P < 0.001). After adjusting for all other factors in the multivariate model, being male, having fever, enlarged spleen and anaemia were all associated with excess odds of infection. Higher odds of infection were observed in all age groups when compared to adults above 30 years, with the highest odd in the 11–15 age group (adjusted odd ratio, AOR = 3.38, P < 0.001). Moreover, living in the coastal mainland (Ungoye) and large island (Mfangano Island) were found to be significantly associated with malaria infection. The odds of malaria infection were almost nine times higher (AOR = 8.55, P < 0.001) for residents of Ungoye and almost four times higher (AOR = 3.46, P < 0.001) for residents of Mfangano when compared to those of Ngodhe. Interestingly, the odds of malaria infections were not significantly different among small islands.

## Discussion

The present study is the first large-scale description of malaria prevalence on islands in Lake Victoria in Kenya based on both microscopy and PCR methods. Our findings indicate significant local variation in malaria prevalence in the study area, a small region of altitudinal and vegetational homogeneity with stable perennial transmission. The relative abundance of vector species and suitable larval habitats likely contribute to this variation in malaria prevalence between neighbouring islands and mainland in Lake Victoria. Entomological studies have revealed that the dominant malaria vector species in all studied islands is *Anopheles gambiae* s.s., but in the past decade it has been replaced by *An. arabiensis* in the coastal mainland of Lake Victoria^8^. On islands, the grazing area for livestock is limited, which may reduce the availability of livestock for the zoophilic *An. arabiensis*^12^, thus suppressing the number of this species. Minakawa *et al*.^13^ reported that availability of *Anopheles* larval habitats found in the late rainy season in Lake Victoria was strongly affected by human activities such as man-made holes and roadside ditches created by vehicles and irrigation. In Ungoye unpaved road and extensive irrigation for farming provide ample favourable *Anopheles* larval habitats, which likely contribute to the high malaria prevalence in this area. Nonetheless, other factors underlying the variation of malaria endemicity on our study area are not fully understood, but may include geomorphological or geophysical formation^14^, household structure features, use of protective measures, variation in distance to the nearest mosquito breeding site^15^, and both human behavioural and genetic factors that may also result in differential attractiveness to mosquitoes and resistance to malaria parasites.

Local variation in malaria prevalence was also observed among communities on the same island. Our study showed that malaria prevalence by PCR in the eight catchment areas on Mfangano Island ranged from 9% to 59%. For the first time, we reported malaria prevalence by PCR on Ringiti Island, a small inhabited satellite island to the west of Mfangano Island. Our results indicate that even among lowland areas along the shore of Mfangano, parasite rates were higher in some communities (Wakula and Wakinga) than others (Mrongo and Ugina). In the highland areas, parasite rates were lower in Kangungu than Gulwe. The latter is connected by a small access road to Wakula, where prevalence was high, suggesting that human movement might yet be another contributing factor to the micro-geographical variation in malaria endemicity.

Both parasite rates and parasitaemia were strongly age-dependent, although in different ways. While mean malaria parasitaemia was highest among children under five years and decreased with age, prevalence of malaria infections rose throughout childhood and only started decreasing in adolescents and adults. These different patterns may be explained by local mosquito biting behaviour and acquisition of immunity. In addition, the reported use of bed net among school-age children was considerably low (33%) in the lakeside region^16^, mostly because these children did not sleep in beds^17^. The increase in parasite prevalence among school-age children likely reflects an increase in exposure to infective mosquito bites. It is now well established that the main determinant of the age distribution of malaria is the development of anti-parasite immunity that restricts the density of asexual parasitaemia which peaks in children less than 5 years old and subsequently declines in an age-dependant manner^18^. Nonetheless, the mechanisms mediating anti-parasite and anti-disease immunity are complex but are thought to include both innate and adaptive immune responses that limit the blood stage of the parasite cycle in the human host^19^.

Convenience sampling approaches are efficient and cost effective, but are more likely to be flawed by selection bias. In this study, surveys often took place in primary schools, meaning that children of school age (6 to 15 years) were disproportionately represented - the age distribution varied slightly depending on the setting (Supplementary Table S1). As these school-aged children had the highest prevalence by both microscopy and PCR (Table 1), over-representation of this age group among our samples likely overestimated the overall malaria prevalence in the study area.

No *P. vivax* infection was found in any of the surveys by either microscopy or PCR, consistent with the absence in many sub-Saharan populations of the Duffy blood-group-antigen, which is required for red blood cell invasion by *P. vivax*^20^. The predominant species in the study area was *P. falciparum*, consistent with previous studies conducted in Mbita sub-county^10^,^21^,^22^. In this study *P. malariae* was the second most prevalent species. By both microscopy and PCR, the majority of *P. malariae* infections were double co-infections with *P. falciparum*. Triple co-infections of *P. falciparum, P. malariae*, and *P. ovale* were more common than *P. malariae* mono-infection (Table 2). Previous studies conducted in Mbita sub-county also reported the presence of *P. malariae* in mono- and mixed species infections by microscopy^10^,^22^. *P. ovale* was recorded as a minor species and reported cases caused by this species were rare as a result of under-diagnosis or low transmission rates^23^,^24^. *P. ovale* is known for its low densities^25^,^26^ which require more sensitive and specific diagnosis. Consistent with this observation, *P. ovale*-specific prevalence by PCR was 13 times higher than that by microscopy in this study. The prevalence of *P. malariae* and *P. ovale* was higher in Ungoye and Mfangano Island than in the small islands. Apart from difference in wide range of ecological factors such as climate and vector-human interactions, these geographical variations could indicate the existence of various species-specific geographical niches. Differential species burden patterns observed among the study areas call for the need to have diagnostic tools or approaches that can facilitate the detection of such non-falciparum malaria cases^27^.

The high proportions of *P. falciparum* gametocyte carriers were observed among *P. falciparum*-infected children from all study areas during the study periods. Previous study by Churcher *et al*. found evidence that within a narrow age band—6 months to 10 years—there may be a positive association between age and the likelihood of transmission^28^. This has been associated with naturally acquired human immune responses against gametocyte antigens that may reduce or prevent the transmission of malaria parasites to mosquitoes^29^,^30^ and may be highly prevalent in young age groups^29^. In contrast with immune responses to the asexual stages of *P. falciparum*, where effective immunity matures with age, the higher gametocyte densities in children induce higher levels of transmission reducing activity^29^. These studies also suggest that the underlying gametocyte prevalence in different settings can vary widely and has an impact on the size of the infectious reservoir^31^,^32^. Nevertheless, as microscopy is not a very sensitive tool for detecting all relevant densities of gametocytes^32^, we expect the true gametocyte prevalence to be much higher than reported here. Indeed, gametocytes are the currency of transmission from human to mosquito for maintaining the malaria cycle. The presence and infectiousness of gametocytes in circulation determines the success of transmission from humans to mosquitoes^33^.

This study also uncovered the extent of asymptomatic malaria in the Lake Victoria basin in Kenya. The vast majority (91.8% by PCR) of malaria infections in the study area were asymptomatic i.e. not accompanied by febrile symptoms. While these asymptomatic infections were most prevalent in children 15 years and under, they were not uncommon in the adult population. As potential gametocyte carriers, this asymptomatic adult population represents an important reservoir for malaria transmission. Many of these asymptomatic infections are present at densities below the limit for microscopic detection and therefore, the use of microscopy as the sole diagnostic method likely leads to an underestimation of the malaria burden. Our study showed that microscopy detected significantly fewer asymptomatic infections when compared to PCR, consistent with several previous findings that molecular-based assays were more sensitive and time-efficient methods for asymptomatic malaria infections^34^,^35^. Our study found 10.2% of the asymptomatic *P. falciparum* infections detected by microscopy were also carrying gametocytes. Previous studies have shown that a proportion of these individuals may play an important role in malaria transmission^36^,^37^,^38^. In moderate and high transmission settings in Africa, long term persistence of infection and prevalent gametocyte carriage have been observed among those with asymptomatic malaria from weeks to months^39^,^40^. This phenomenon has been explained by a form of acquired immunity that suppresses parasitaemia in low level without achieving complete clearance^18^.

The increase in the proportion of sub-microscopic infections by age was contrasted by the decreases in infection prevalence and parasite densities in the population. Parasite density is mediated by host acquired immunity^37^, in that individuals with higher levels of immunity (i.e. older ages) would maintain parasites at lower densities^41^. Okell *et al*.^42^ reported that the proportion of sub-microscopic infections is inversely correlated with slide prevalence and parasite density on the global level^42^. The majority of sub-microscopic malaria infections in the Lake Victoria area were asymptomatic. The importance of the sub-microscopic asymptomatic parasite pool rests on the understanding that sub-microscopic infections can transmit malaria^43^,^44^,^45^, although the minimum parasite density necessary for transmission is unknown. In the Lake Victoria area, asymptomatic and sub-microscopic infections in older children and adults may be an important source of the local transmission.

Conventional PCR is typically able to detect infections down to approximately one parasite/μl using dried blood spots^43^. Presumably there are many infections below this threshold that are undetectable. Whether to screen and treat using a more sensitive tool or to institute mass drug administration (MDA) without screening requires a better understanding of the minimum density of malaria parasites that results in human-mosquito transmission, as well as the comparative costs and operational ease of different approaches^43^.

Islands present a unique opportunity to interrupt malaria transmission due to the relative geographical isolation and confined population. MDA was previously recognized as an important method of malaria control^46^, and it is thought that malaria can be eliminated on isolated islands using MDA and vector control if there is a high level of community commitment^47^. In the Southwest Pacific, malaria was successfully eliminated by MDA from small islands such as Aneityum in Vanuatu^47^ and Nissan in Papua New Guinea^48^, where population movement was closely monitored and ports of entry were controlled to minimize parasite re-importation. The main challenges for MDA include the need to achieve high treatment coverage, and the necessity to repeat the intervention or combine it with other interventions such as vector control in all endemic settings for long-term effect^49^. As most infections in our study area were asymptomatic and sub-microscopic, more sensitive and field-friendly diagnostic tools are needed to inform timely adjustment to MDA protocols. Furthermore, robust surveillance systems to detect malaria resurgence after successful MDA or elimination may be crucial. A number of studies have indicated that while semi-immunity to malaria can be acquired in highly endemic areas by the age of 5 years, this immunity wanes rapidly without ongoing parasite exposure^18^,^50^. In the event of resurgence, children and adolescents might be more susceptible to reinfection and development of clinical and severe malaria due to their shorter duration of previous exposure to parasites when compared to adults.

In summary, this study highlighted the heterogeneity in malaria prevalence and importance of asymptomatic and sub-microscopic infections on islands and a coastal area in Lake Victoria. We observed significant geographical differences and age-dependency in malaria parasite carriage. The unique findings of malaria epidemiology in our study area may have important implications for the development and implementation of malaria elimination strategy. Taking into account the high and heterogeneous prevalence of asymptomatic and sub-microscopic malaria infections on islands in Lake Victoria, the use MDA to eliminate malaria may be justified. An appropriate package that complements MDA with effective vector control and rapid detection and treatment of new cases has the potential to achieve long-term malaria elimination on islands in Lake Victoria.

## Methods

### Ethics statement

The study was conducted in accordance with the Declaration of Helsinki and was approved by the Kenyatta National Hospital/University of Nairobi-Ethics and Research Committee in Kenya (No. P7/1/2012) and the Committee on the Ethics of Human Research of Karolinska Institutet in Sweden (Dnr 201271239–31/4). Survey subjects were informed by local interpreters of the purposes and procedures of the study, and a written informed consent was obtained from each adult participant at study registration. In the case of children, written informed consents were obtained from their parents or legal guardians.

### Study area and population

**Figure 6:**
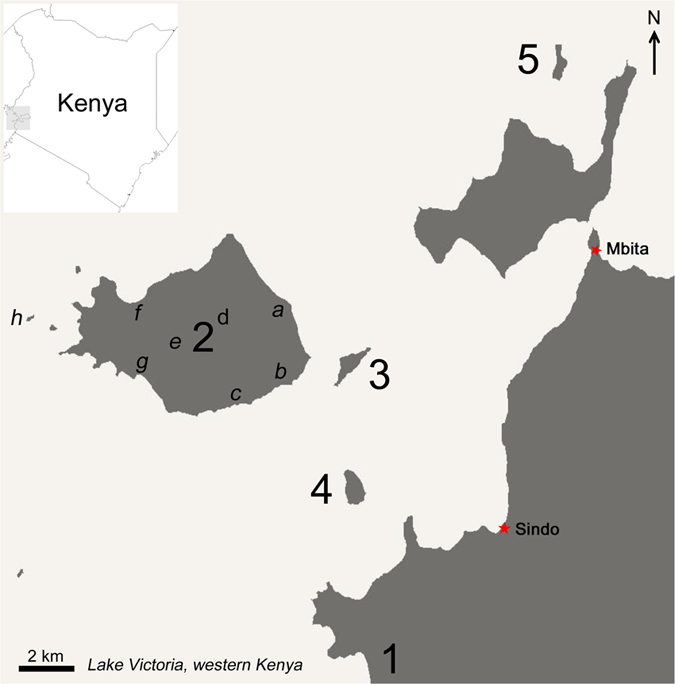
Map of study setting in Lake Victoria, Homabay County in western Kenya where malaria surveillances were conducted. Three main areas involved in the five surveys namely (1) mainland coast (Ungoye), (2) large island (Mfangano Island) and small islands [(3) Takawiri, (4) Kibuogi and (5) Ngodhe)]. In large island of Mfangano, eight catchment points were sampled and grouped into three study sites i.e. east coast [(*a*) Ramba, (*b*) Wakinga and (*c*) Mrongo], highland [(*d*) Kagungu and (*e*) Gulwe] and west coast [(*f*) Wakula, (*g*) Ugina and (*h*) Ringiti Island]. The map was created with ArcGIS software, version 10.4, http://www.esri.com.

This study was conducted in Homa Bay County, Kenya and included one large island (Mfangano) and three small islands (Ngodhe, Kibuogi and Takawiri) in Lake Victoria, and one coastal village (Ungoye) on the mainland (Fig. 6). The dominant ethnic group in the study area is Luo; Dholuo is primarily spoken, as well as the national language of Kiswahili.

In western Kenya a bimodal pattern of rainfall is generally observed, with the long rainy season from March to June and the short rainy season from November to December, but the periods vary each year. Annual rainfall ranges from 700 mm to 1,200 mm. The mean temperature is 25 °C while the maximum temperature is 30 °C, and humidity is relatively high. Malaria prevalence peaks usually lag 1–2 months after the rainy seasons. In the early 1990 s, annual entomological inoculation rates (EIRs) reported between 60 and 300 infectious bites per person per year^51^,^52^. Nevertheless, following the distribution of insecticide-treated net (ITN), the EIR equivalents estimated from serological markers were reduced to fewer than 50 infectious bites per person per year^53^. The important malaria vectors in this region are *An. gambiae* s.s., *An. arabiensis* and *An. funestus*^13^. *An. gambiae* s.s. has experienced a reduction in density of nearly 95% in the Lake Victoria basin over 10 years, and has been replaced by *An. arabiensis* as the dominant malaria vector species in the mainland part of this region. Nevertheless, *An. gambiae* s.s is still the predominant malaria vector on islands in Lake Victoria^8^ and its abundance can increase up to eight-fold during the long rainy season^14^. HIV and other parasitic infections such as schistosomiasis and filariasis are highly endemic in this area^54^.

Though there are many development initiatives in these areas, poverty is still a major challenge. Almost half of the population in Homa Bay County survives on below the Kenyan poverty line of Kenya Shilling (KSh) 1,562 (USD 15.6) per month^55^. Farming and fishing are the major economic activities for the island and lakeshore communities. Farming is mostly subsistence-based, with major crops including maize, millet and beans as well as small-scale animal husbandry (cattle, goats and chickens). Fishing is done primarily by men using small, unpowered wooden boats. Other fishing related activities such as registration of fishermen and fishing vessels, and sale and purchase of fish are coordinated by community-based beach management units (BMU). Houses are typically made of mud walls with thatched or corrugated iron roofs.

### Large Island: Mfangano

Lying just east of the border with Uganda, Mfangano (Fig. 6; 66 km^2^) has a population of approximately 18,600 (HDSS). The island’s terrain is rocky and hilly. The island is easily recognisable from the mainland by the imposing green-clad cliffs of Mount Kwitutu, which rises 562 m above the lake surface from the island’s centre. Mfangano Island is predominantly rural. Nevertheless, it is well connected to Mbita (16 km) in the mainland by a popular ferry service and numerous private, scheduled and unscheduled motorized boat trips. Residents of Mfangano Island rely on the neighbouring Mbita for regional transportation and access to services such as banks. There are six government health facilities (one health centre and five dispensaries) across the island. In contrast to other study sites, on Mfangano there is a sizable Suba speaking population which is becoming rarer due to the intermarriage between the Suba and Luo ethnic tribes. In addition to farming and fishing, tourism is also growing on this island as the connection to and from Mbita is being improved.

There were eight catchment points on Mfangano: Ramba, Wakinga and Mrongo along the east coast (HDSS, population 1642), Kagungu and Gulwe in the highland (HDSS, population 827), and Wakula, Ugina, and Ringiti Island in the west (HDSS, population 1936). These catchment points were selected to represent the different local environments found on the island, from the lakeshore lowland areas to the central highland areas.

### Small Islands: Ngodhe, Kibuogi and Takawiri

Each of these three small islands (Fig. 6) has a population of approximately 700 (HDSS) distributed between two main settlements or boat landing beaches: Bonde and Luanda on Ngodhe Island, Kibuogi A and Kibuogi B on Kibuogi Island, and Kamarach and Kongata on Takawiri Island. The islands’ elevation is up to 70 m from the lake level. Takawiri and Kibuogi are close to Mfangano and the boat traffic connecting among these islands is frequent. The distances between Mbita in the mainland and Takawiri and Kibuogi are approximately 14 km and 16 km, respectively. On the other hand, Ngodhe Island is situated to the north of Rusinga Island, which is connected to Mbita via a causeway. These islands have been extensively deforested and shrubs constitute the main vegetation today. Takawiri and Ngodhe are each served by a dispensary, but no public health facility is available on Kibuogi.

### Coastal mainland: Ungoye

Ungoye (Fig. 6) is a small village (19.3 km^2^) in Nyangwethe area with population of 3,471 (2009 census). The village is connected by an unpaved roadway to the nearby small towns of Sindo (17.2 km) and Mbita Point (31.2 km), each with a sub-district hospital. There is one small public health facility in this area. Ungoye was included for comparative purposes due to its similarity to the islands in environmental characteristics, infrastructure, and access to health services. A recent survey in the Gembe East area revealed a high *P. falciparum* prevalence of 66.5% by PCR^56^.

### Study design and data collection

Five cross-sectional surveys were conducted between 2012 and 2014: two after the short rainy season in January-February 2012 and March 2014 and three after the long rainy season in July-August 2012, August 2013, and August 2014. Peak malaria transmission occurs 1–2 months after the rainy season and the mean monthly anopheline vector abundance has been reported to increase by 6- to 8-fold in the rainy season compared to the dry season in the Lake Victoria basin^14^; thus, surveys were conducted approximately 2 months after the rainy seasons to coincide with the periods of heaviest malaria burden. All sites were included in each cross-sectional survey, with the exception that Mfangano and Takawiri were not included in the March 2014 survey. This study used the convenience sampling strategy, whereby residents were asked to come to selected survey points such as BMU community hall, school or market place for study participation. During this three-year period, we did not see any major changes or events (e.g. mass distribution of ITNs and indoor residual spraying in previously uncovered areas) which might an impact on our study findings.

### Field and laboratory methods

Sex and age were recorded for each participant. Axillary body temperature was determined using a digital thermometer (Terumo, New Jersey, US). Fever was defined as a temperature exceeding 37.5 °C. Hemoglobin level was measured with the HemoCue Hb 201 analyzer (HemoCue, Angelholm, Sweden). A measurement below 11 g/dL was classified as anaemic. Children aged 12 years and below were examined for enlarged spleen by AK according to Hackett’s method, regardless of fever or malaria status. Measuring spleen sizes in children has been shown as a useful variable to estimate the burden of malaria transmission in other island settings^57^.

Thin and thick blood smears were prepared on site, stored in slide boxes and transported daily to the main laboratory in Mbita, where thin blood smears were fixed with methanol, and all smears were stained with 3% Giemsa solution for 30 minutes and examined under oil emersion (10 × 100 magnification) by experienced microscopists. Blood smears were defined as negative if no parasites were found after examining 100 high power microscopy fields. For all positive samples, malaria species were identified and asexual parasite forms were counted against 200 leukocytes. *P. falciparum* gametocytes counts were separately recorded. Parasite density was estimated from parasite counts, assuming that there were 8,000 leukocytes per μl of blood. All slides were independently re-examined by two experienced microscopists blinded to the first microscopy reading results. Discrepancies between the two readings were resolved by a third experienced microscopist.

For detection by PCR, blood samples (70 μl) withdrawn by 75 mm Micro-hematocrit capillary tube (Thermo Fisher Scientific, MA, USA) were spotted on Whatman ET31 Chr filter papers (Whatman International, Maidstone, UK) and dried thoroughly at ambient temperature. The dried blood spots were put in individual zipped plastic bags and stored at −20 °C. DNA was extracted from a quartered blood spot (17.5 μl) using the QIAamp Blood Mini Kit (QIAGEN, Germantown, USA) according to the manufacturer’s instructions. *Plasmodium* infection was detected by a nested PCR protocol that can distinguish the four major human malaria species (except *P. knowlesi*)^58^.

### Data analysis

All survey data were double entered into Microsoft Excel spreadsheets and cross-checked for errors. Data were processed and analysed using the STATA/SE 13.1 statistical software package (StataCorp, TX, USA). Differences in participant characteristics and prevalence among age groups and geographical areas were assessed using Chi-squared (χ^2^) or Fisher’s exact tests. The average enlarged spleen (AES) index for each survey was calculated as the sum of the number of children in each spleen size class multiplied by the class number (0–4), divided by the total number of palpable spleen. Interactions between survey, individual settings, age group and prevalence were examined using conditional logistic regression. Univariate logistic regression was performed to identify risk factors for the outcome of *Plasmodium* infection as determined by PCR. Sex, age group, geographical area, fever, enlarged spleen and anaemia were considered as individual variables. All variables with a P-value of less than 0.05 from a likelihood ratio test in univariate analyses were entered into a multivariate logistic regression model and stepwise backwards elimination was used to identify the main risk factors for infection.

## Additional Information

**How to cite this article**: Idris, Z. M. *et al*. High and Heterogeneous Prevalence of Asymptomatic and Sub-microscopic Malaria Infections on Islands in Lake Victoria, Kenya. *Sci. Rep.* **6**, 36958; doi: 10.1038/srep36958 (2016).

**Publisher’s note**: Springer Nature remains neutral with regard to jurisdictional claims in published maps and institutional affiliations.

## Supplementary Material

Supplementary Information

## Figures and Tables

**Table 1:** Prevalence of malaria infection in five surveys by microscopy and PCR.

Attributes	Category	January - February 2012, N (%) [95% CI]		July - August 2012, N (%) [95% CI]		August 2013, N (%) [95% CI]		March 2014, N (%) [95% CI]		August 2014, N (%) [95% CI]		Overall, N (%) [95% CI]	
		Parasite positive		Parasite positive		Parasite positive		Parasite positive		Parasite positive		Parasite positive	
		Microscopy	PCR	Microscopy	PCR	Microscopy	PCR	Microscopy	PCR	Microscopy	PCR	Microscopy	PCR
Overall parasite rate*		2577 (18.7)[17.2–20.2]	2586 (39.3)[37.4–41.2]	2665 (14.6)[13.3–16.0]	2654 (30.1)[28.4–31.9]	2246 (22.5)[20.8–24.3]	2253 (39.1)[37.0–41.1]	771 (24.1)[21.1–27.3]	765 (44.2)[40.6–47.8]	2172 (23.6)[21.8–25.5]	2172 (38.4)[36.4–40.5]	10431 (19.9)[19.1–20.7]	10430 (37.1)[36.2–38.0]
**Gender**§	**Male**	1205 (22.1)[19.8–24.5]	1208 (44.1)[41.3–47.0]	1306 (14.9)[13.0–17.0]	1299 (31.9)[29.3–34.5]	1085 (23.0)[20.6–25.7]	1089 (39.9)[37.0–42.9]	385 (22.3)[18.3–26.8]	382 (44.8)[39.7–49.9]	1003 (24.5)[21.9–27.3]	1003 (39.9)[36.8–43.0]	4984 (20.9)[19.8–22.1]	4981 (39.2)[37.8–40.6]
	**Female**	1372 (15.7)[13.8–17.7]	1378 (35.1)[32.6–37.7]	1357 (14.3)[12.5–16.3]	1353 (28.5)[26.1–30.9]	1160 (22.1)[19.7–24.6]	1163 (38.2)[35.4–41.0]	386 (25.9)[21.6–30.6]	383 (43.6)[38.6–48.7]	1168 (22.9)[20.5–25.4]	1168 (37.2)[34.4–40.1]	5443 (19.0)[17.9–20.0]	5445 (35.2)[33.9–36.5]
**Age group (years)**	**0–5**	638 (23.5)[20.3–27.0]	639 (38.0)[34.2–41.9]	635 (16.1)[13.3–19–2]	631 (28.7)[25.2–32.4]	562 (26.5)[22.9–30.4]	565 (33.6)[29.7–37.7]	189 (33.3)[26.7–40.5]	187 (56.2)[48.7–63.4]	501 (24.6)[20.8–28.6]	501 (37.9)[33.7–42.3]	2525 (23.3)[21.6–24.9]	2523 (36.0)[34.2–37.9]
	**6–10**	642 (24.1)[20.9–27.6]	645 (49.0)[45.1–52.9]	756 (21.3)[18.4–24.4]	757 (36.6)[33.2–40.1]	523 (35.4)[31.3–39.6]	524 (48.1)[43.7–52.5]	291 (33.0)[27.6–38.7]	290 (50.3)[44.4–56.2]	597 (26.8)[23.3–30.5]	597 (40.9)[36.9–44.9]	2809 (27.0)[25.3–28.6]	2813 (43.9)[42.1–45.8]
	**11–15**	705 (18.9)[16.0–22.0]	708 (50.0)[46.3–53.7]	478 (15.9)[12.7–19.5]	472 (38.1)[33.7–42.7]	403 (29.3)[24.9–34.0]	405 (57.8)[52.8–62.6]	118 (17.8)[11.4–25.9]	118 (46.6)[37.4–56.0)	459 (32.9)[28.6–37.4]	459 (50.3)[45.7–55.0]	2163 (23.1)[21.3–24.9]	2162 (48.8)[46.6–50.9]
	**16–30**	318 (6.9)[4.4–10.3]	318 (18.2)[14.2–22.9]	457 (7.4)[5.2–10.2]	455 (23.3)[19.5–27.5]	408 (8.8)[6.3–12.0]	409 (28.1)[23.8–32.7]	79 (6.3)[2.1–14.2]	76 (23.7)[14.7–34.8]	316 (13.9)[10.3–18.2]	316 (29.8)[24.8–35.1]	1578 (8.9)[7.6–10.5]	1574 (24.8)[22.7–27.1]
	**>30**	272 (7.7)[4.8–11.6]	274 (16.4)[12.2–21.4]	339 (4.7)[2.7–7.6]	339 (16.2)[12.5–20.6]	349 (5.2)[3.1–8.0]	349 (25.2)[20.7–30.1]	94 (1.1)[0.0–5.7]	94 (14.9)[8.4–23.7]	298 (11.7)[8.3–16.0]	298 (25.5)[20.7–30.8]	1352 (6.7)[5.5–8.2]	1354 (20.5)[18.4–22.8]
**Setting**	**Ungoye**	622 (35.2)[31.5–39.1]	622 (58.2)[54.2–62.1]	616 (29.1)[25.5–32.8]	615 (58.1)[54.0–62.0]	716 (46.9)[43.2–50.7]	720 (71.1)[67.6–74.4]	317 (50.5)[44.8–56.1]	314 (79.3)[74.4–83.6]	543 (40.5)[36.4–44.8]	543 (54.5)[50.2–58.8]	2814 (39.6)[37.8–41.4]	2814 (63.1)[61.3–64.9]
	**Mfangano**	883 (23.3)[20.6–26.3]	890 (55.5)[52.2–58.8]	1118 (15.1)[13.1–17.4]	1112 (29.7)[27.0–32.5]	788 (16.9)[14.3–19.7]	790 (34.1)[30.7–37.5]	nd	nd	906 (23.1)[20.4–26.0]	906 (43.4)[40.1–46.7]	3695 (19.4)[18.1–20.7]	3698 (40.2)[38.6–41.8]
	**Takawiri**	599 (4.3)[2.9–6.3]	601 (16.3)[13.4–19.5]	435 (2.5)[1.3–4.5]	432 (9.7)[7.1–12.9]	285 (7.7)[4.9–11.5]	286 (14.7)[10.8–19.3]	nd	nd	362 (11.1)[8.0–14.7]	362 (19.6)[15.6–24.1]	1681 (5.9)[4.8–7.1]	1681 (15.1)[13.4–16.9]
	**Kibuogi**	130 (9.2)[4.9–15.6]	130 (26.9)[19.5–35.4]	206 (4.4)[2.0–8.1]	206 (10.2)[6.4–15.2]	204 (3.4)[1.4–6.9]	204 (10.3)[6.5–15.3]	211 (4.3)[2.0–7.9]	209 (12.0)[7.9–17.1]	149 (9.4)[5.2–15.3]	149 (21.5)[15.2–28.9]	900 (5.7)[4.2–7.4]	898 (14.9)[12.7–17.4]
	**Ngodhe**	343 (5.2)[3.1–8.2]	343 (8.2)[5.5–11.6]	290 (7.2)[4.5–10.9]	289 (17.0)[12.8–21.8]	253 (3.2)[1.4–6.1]	253 (14.2)[10.2–19.2]	243 (7.0)[4.1–11.0]	242 (26.5)[21.0–32.5]	212 (14.2)[9.8–19.6]	212 (20.3)[15.1–26.3]	1341 (7.0)[5.7–8.5]	1339 (16.4)[14.5–18.5]
**Geometric mean parasite count (/μL) (min-max)**		922 (40–196332)		1372 (39–71800)		1557 (40–368440)		1197 (40–54200)		874 (38–100000)		1143 (1060–1232)	

^*^Total of 33 individuals has no data on microscopy (Jan. 2012; 12, Aug. 2012; 3, Aug. 2013; 17).

^*^Total of 14 individuals has no data for PCR (Aug. 2012) and 28 samples were missing for PCR (Jan. 2012; 4, Aug. 2012; 3, Aug. 2013; 15, Mar. 2014; 6).

^§^No gender data but negative: 8 by microscopy (Aug. 2012; 2, Aug. 2013; 5, Aug. 2014; 1) and 11 by PCR (Aug. 2012; 2, Aug. 2013; 8, Aug. 2014; 1). One positive by PCR (Aug. 2013).

No age data but negative: 4 by microscopy (Jan. 2012; 2, Aug. 2013; 1, Aug. 2014; 1), and 2 by PCR (Jan. 2012; 1, Aug. 2014; 1). Two positive by PCR (Jan. 2012; 1, Aug. 2013; 1). nd = not done, CI = confidence interval.

**Table 2:** Overall *Plasmodium* spp. and gametocyte prevalence by microscopy and PCR in Lake Victoria.

Diagnosis method	N	Positive microscopy, % (95% CI)	Positive PCR, % (95% CI)	Prevalence of infection								
				Pf	Pm	Po	Pv	Pf+Pm	Pf+Po	Pm+Po	Pf+Pm+Po	Pf gametocyte
**Microscopy**	10431	19.9 (19.1–20.7)		1913	19	2	0	110	25	1	6	211
**PCR**	10430		37.1 (36.2–38.0)	2489	133	71	0	760	147	7	262	

N = total number of participants; Pf: *P. falciparum*; Pm: *P. malariae*; Po: *P. ovale*; Pv: *P. vivax*; CI = confidence interval.

**Table 3:** Proportion of populations with gametocytes in all surveys detected by microscopy.

Variable	Category	N	n (%)[95% CI]
**Age group (years)**	**0–5**	576	80 (13.9)[11.1–17.0]
	**6–10**	755	86 (11.4)[9.2–13.9]
	**11–15**	490	32 (6.5)[4.5–9.1]
	**16–30**	141	8 (5.7)[2.5–10.9]
	**>30**	91	5 (5.5)[1.8–12.4]
**Setting**	**Ungoye**	1108	111 (10.0)[8.3–11.9]
	**Mfangano**	704	73 (10.4)[8.2–12.9]
	**Takawiri**	98	10 (10.2)[5.0–18.0]
	**Kibuogi**	51	10 (19.6)[9.8–33.1]
	**Ngodhe**	92	7 (7.6)[3.1–15.1]

N = total number of participants with *P. falciparum* infections by microscopy; n = number of individuals positive for gametocytes parasite; CI = confidence interval.

**Table 4:** Risk factor for *Plasmodium* infection identified by PCR analyses.

Risk factor	Category	N	PCR positivity rate, % (95% CI)	COR (95% CI)	P-value	AOR (95% CI)	P-value
**Gender**	**Male**	4981	39.2 (37.8–40.6)	1.19 (1.10–1.29)	<0.001	1.21 (1.11–1.32)	<0.001
	**Female**	5445	35.2 (33.9–36.5)	1		1	
**Febrile (>37.5 **°**C)**	**Yes**	577	54.8 (50.6–58.9)	2.15 (1.81–2.54)	<0.001	1.79 (1.49–2.16)	<0.001
	**No**	9848	36.1 (35.1–37.0)	1		1	
**Enlarge spleen (≤12 year olds)**	**Yes**	2545	71.6 (69.8–73.4)	7.46 (6.67–8.34)	<0.001	5.79 (5.11–6.56)	<0.001
	**No**	4003	25.3 (23.9–26.7)	1		1	
**Anaemia (<11 g/dL)**	**Yes**	2290	41.8 (39.8–43.9)	1.29 (1.17–1.42)	<0.001	1.29 (1.16–1.43)	<0.001
	**No**	8127	35.8 (34.7–36.8)	1		1	
**Age group (years)**	**0–5**	2523	36.0 (34.2–37.9)	2.18 (1.87–2.55)	<0.001	1.70 (1.43–2.01)	<0.001
	**6–10**	2813	43.9 (42.1–45.8)	3.03 (2.60–3.52)	<0.001	2.76 (2.35–3.24)	<0.001
	**11–15**	2162	48.8 (46.6–50.9)	3.68 (3.15–4.31)	<0.001	3.38 (2.86–3.98)	<0.001
	**16–30**	1574	24.8 (22.7–27.1)	1.28 (1.07–1.52)	0.006	1.35 (1.13–1.62)	0.001
	**>30**	1354	20.5 (18.4–22.8)	1		1	
**Setting**	**Ungoye**	2814	63.1 (61.3–64.9)	8.70 (7.39–10.25)	<0.001	8.55 (7.24–10.10)	<0.001
	**Mfangano**	3698	40.2 (38.6–41.8)	3.42 (2.92–4.01)	<0.001	3.46 (2.95–4.06)	<0.001
	**Takawiri**	1681	15.1 (13.4–16.9)	0.90 (0.74–1.10)	0.3	0.85 (0.69–1.03)	0.1
	**Kibuogi**	898	14.9 (12.7–17.4)	0.89 (0.71–1.12)	0.338	0.87 (0.68–1.10)	0.231
	**Ngodhe**	1339	16.4 (14.5–18.5)	1		1	

Variable listed are those which were retained in the final multivariate model, with both the output from univariate and multivariate models presented with odd ratios (OR) and 95% confidence intervals (95% CI), as well as likelihood ratio test P-values. N = sample size, COR = crude odd ratios (predicted difference in odds of malaria infection compared to the baseline odds of malaria infection in the reference group), AOR = adjusted odd ratios (predicted difference in odds of malaria infection adjusting among all the explanatory variables), CI = confidence interval.
