# Supplementary material for: High and Heterogeneous Prevalence of Asymptomatic and Sub-microscopic Malaria Infections on Islands in Lake Victoria, Kenya
Source: Sci Rep. 2016 Nov 14;6:36958. doi: 10.1038/srep36958 (PMC5107902; doi:10.1038/srep36958)
Supplement: Supplementary Information [file srep36958-s1.pdf]

Legend supplementary data

High and Heterogeneous Prevalence of Asymptomatic and Sub-microscopic Malaria Infections on Islands in Lake Victoria, Kenya

Zulkarnain Md Idris[1, 2], Chim W. Chan[1], James Kongere[3], Jesse Gitaka[4], John Logedi[5], Ahmeddin Omar[5], Charles Obonyo[6], Beatrice Kemunto Machini[5], Rie Isozumi[7], Isao Teramoto[7], Masatsugu Kimura[7], Akira Kaneko[1, 7, 8*]

[1] Island Malaria Group, Department of Microbiology, Tumor and Cell Biology, Karolinska Institutet, Stockholm, 17177, Sweden

[2] Department of Parasitology and Medical Entomology, Faculty of Medicine, Universiti Kebangsaan Malaysia Medical Centre, Kuala Lumpur, 56000, Malaysia

[3] Nagasaki University Nairobi Research Station, NUITM-KEMRI Project, Nairobi, 00202, Kenya

[4] Department of Clinical Medicine, Mount Kenya University, Thika, 01000, Kenya

[5] National Malaria Control Programme, Ministry of Public Health, Nairobi, 00100, Kenya

[6] Kenya Medical Research Institute (KEMRI), Centre for Global Health Research, Kisumu, 40100, Kenya

[7] Department of Parasitology, Graduate School of Medicine, Osaka City University, Osaka, 558-8585, Japan

[8] Institute of Tropical Medicine, Nagasaki University, Nagasaki, 852-8102, Japan

*corresponding.akira.kaneko@ki.se

Supplementary Table S1 **Demographic and clinical characteristics of the study population, by survey.**

Supplementary Table S2 **Univariate associations with malaria parasite density estimate by microscopy.**

Supplementary Table S3 **Proportion of individual with sub-microscopic infection (PCR positive but microscopy negative) by setting in all surveys.**

Supplementary Table S1

Attributes	Category	Total N = 10450		January-February 2012 N = 2586		July-August 2012 N = 2668		August 2013 N = 2253		March 2014 N = 771		August 2014 N = 2172	
		n	% (95% CI)	n	% (95% CI)	n	% (95% CI)	n	% (95% CI)	n	% (95% CI)	n	% (95% CI)
**Demographic characteristics**													
Gender*	Male	4993	47.8 (46.8-48.7)	1208	46.7 (44.8-48.7)	1308	49.1 (47.4-51.0)	1089	48.4 (46.3-50.4)	385	49.9 (46.3-53.5)	1003	46.2 (44.1-48.3)
	Female	5453	52.2 (51.2-53.1)	1378	53.3 (51.3-55.2)	1358	50.9 (49.0-52.9)	1163	51.6 (49.6-53.7)	386	50.1 (46.5-53.7)	1168	53.8 (517-55.9)
Median age (years, IQR)		10 (6 - 19)		11 (6 - 15)		10 (6 - 20)		11 (5 - 22)		9 (6 - 14)		10 (6 - 20)	
Age group (years)**	0 - 5	2530	24.2 (23.4-25.1)	639	24.7 (23.1-26.4)	636	23.8 (22.2-25.5)	565	25.1 (23.3-26.9)	189	24.5 (21.5-27.7)	501	23.1 (21.3-24.9)
	6 - 10	2814	26.9 (26.1-27.8)	645	25.0 (23.3-26.7)	757	28.4 (26.7-30.1)	524	23.3 (21.5-25.1)	291	37.7 (34.3-41.3)	597	27.5 (25.6-29.4)
	11 - 15	2168	20.8 (20.0-21.5)	708	27.4 (25.7-29.2)	478	17.9 (16.5-19.4)	405	18.0 (16.4-19.6)	118	15.3 (12.8-18.0)	459	21.1 (19.4-22.9)
	16 - 30	1580	15.1 (14.4-15.8)	318	12.3 (11.1-13.6)	458	17.2 (15.8-18.7)	409	18.2 (16.6-19.8)	79	10.3 (8.2-12.6)	316	14.6 (13.1-16.1)
	>30	1354	13.0 (12.3-13.6)	274	10.6 (9.4-11.9)	339	12.7 (11.5-14.0)	349	15.5 (14.0-17.1)	94	12.2 (10.0-14.7)	298	13.8 (12.3-15.2)
Setting													
Coast	Ungoye	2819	26.9 (26.1-27.8)	622	24.1 (22.4-25.7)	617	23.1 (21.5-24.8)	720	32.0 (30.0-33.9)	317	41.1 (37.6-44.7)	543	25.0 (23.2-26.9)
Large island	Mfangano	3706	35.5 (34.5-36.4)	890	34.4 (32.6-36.3)	1120	42.0 (40.1-43.9)	790	35.1 (33.1-37.1)	nd	nd	906	41.7 (36.6-43.8)
Small islands	Takawiri	1684	16.1 (15.4-16.8)	601	23.2 (21.6-24.9)	435	16.3 (14.9-17.8)	286	12.7 (11.3-14.1)	nd	nd	362	16.7 (15.1-18.3)
	Kibuogi	900	8.6 (8.1-9.2)	130	5.0 (4.2-5.9)	206	7.7 (6.7-8.8)	204	9.0 (7.9-10.3)	211	27.4 (24.2-30.7)	149	6.9 (5.8-8.0)
	Ngodhe	1341	12.8 (12.2-13.5)	343	13.3 (12.0-14.6)	290	10.9 (9.7-12.1)	253	11.2 (10.0-12.6)	243	31.5 (28.2-34.9)	212	9.7 (8.5-11.1)
**Clinical characteristics**													
Measured fever (axillary >37.5^{o}C)		10445	5.5 (5.1-6.0)	2586	6.8 (5.8-7.7)	2666	4.8 (4.0-5.6)	2251	4.1 (3.3-4.9)	771	10.4 (8.2-12.5)	2171	4.8 (3.9-5.7)
Enlarged spleen (children ≤12 year olds)		6561	38.9 (37.7-40.1)	1676	36.4 (34.1-38.7)	1554	44.7 (42.2-47.1)	1479	36.9 (34.4-39.3)	539	47.1 (42.9-51.4)	1313	34.2 (31.6-36.8)
Hackett's grade for enlarged spleen	0 (not enlarged)	4009	61.1 (59.9-62.3)	1066	63.6 (61.2-65.9)	860	55.3 (52.8-57.8)	934	63.2 (60.6-65.6)	285	52.9 (48.7-57.2)	864	65.8 (63.2-68.4)
	1	1116	17.0 (16.1-17.9)	292	17.4 (15.6-19.3)	289	18.6 (16.7-20.6)	241	16.3 (14.4-18.3)	92	17.1 (14.0-20.5)	202	15.4 (13.5-17.5)
	2	1114	17.0 (16.1-17.9)	267	15.9 (14.2-17.8)	310	20.0 (18.0-22.0)	236	16.0 (14.1-17.9)	116	21.5 (18.1-25.2)	185	14.1 (12.3-16.1)
	3	304	4.6 (4.1-5.2)	50	3.0 (2.2-3.9)	91	5.9 (4.7-7.1)	63	4.3 (3.3-5.4)	41	7.6 (5.5-10.2)	59	4.5 (3.4-5.8)
	4	18	0.3 (0.2-0.4)	1	0.1 (0.0-0.3)	4	0.3 (0.1-0.7)	5	0.3 (0.1-0.8)	5	0.9 (0.3-2.2)	3	0.2 (0.0-0.7)
Anaemia (<11 g/dL)		10437	22.0 (21.2-22.8)	2586	20.7 (19.2-22.3)	2663	20.7 (19.1-22.2)	2249	23.1 (21.3-24.8)	770	28.8 (25.6-32.0)	2169	21.6 (19.8-23.3)

N = number of participants sampled, n = number of participants tested, nd = not done, IQR = interquartile range, CI = confidence interval
Not all participants were recorded for each parameter, therefore percentage were calculated based on the number of available in each survey
*4 individual with no gender recorded (Aug. 2012; 2, Aug. 2013; 1, Aug. 2014; 1)
**4 individual with no age recorded (Jan. 2012; 2, Aug. 2013; 1, Aug. 2014; 1)

Supplementary Table S2

Attributes	Category	n	Geometric mean of parasite density (parasite/µL)	Regression coefficients	95% CI	P-value
**Demographic characteristics**						
Age group (years)	0 - 5	570	2023		Ref	
	6 - 10	743	1374	-5624.04	-7494.9, -3754.2	<0.001
	11 - 15	490	675	-7625.69	-9695.6, -5555.8	
	16 - 30	135	494	-6405.71	-9621.8, -3189.6	
	>30	90	412	-8147.03	-111958.1, -4335.9	
Setting	Ungoye	1091	1114		Ref	
	Mfangano	700	1141	-704.71	-2356.4, 946.9	0.589
	Takawiri	95	1257	-1018.47	-4666.9, 2629.9	
	Kibuogi	50	1671	989.37	- 3943.2, 5922.0	
	Ngodhe	92	1152	2124.1	-1578.6, 5826.8	
**Clinical Characteristics**						
Measured fever (axillary>37.5^{o}C)	No	1817	1014		Ref	
	Yes	211	3188	12534.13	10114.6, 14953.7	<0.001
Enlarged spleen (children ≤12 year olds)	No	482	893		Ref	
	Yes	1157	1659	197.65	-1680.3, 2075.6	0.837
Hackett's grade for enlarged spleen	0	482	893		Ref	
	1	362	1234	-1350.10	-3754.4, 1054.2	0.044
	2	582	1705	40.76	-2088.3, 2169.8	
	3	204	2534	3443.27	555.8, 6330.7	
	4	9	2940	-969.69	-12599.9, 10660.5	
Anaemia (<11 g/dL)	No	1421	863		Ref	
	Yes	606	2201	5413.79	3775.7, 7051.9	<0.001

n = number of participant with parasite count, CI = confidence interval

Supplementary Table S3

Variable	Category	N	n	% (95% CI)	P-value
**Setting**	Ungoye	1771	735	41.5 (39.2 - 43.8)	<0.001
	Mfangano	1480	821	55.5 (52.9 - 58.0)	
	Takawiri	253	165	65.2 (59.0 - 71.1)	
	Kibuogi	134	85	63.4 (54.7 - 71.6)	
	Ngodhe	221	140	63.3 (56.6 - 69.7)	

N = total number of microscopic and sub-microscopic individuals, n = number of individuals with sub-microscopic infections, CI = confidence interval
